# A combined approach using environmental yeasts and microbial indicators to assess aquatic pollution

**DOI:** 10.1007/s10661-026-15445-4

**Published:** 2026-05-25

**Authors:** Andressa Alves Silva Panatta, Jéssyca Ketterine Carvalho, Rosemeire Aparecida Silva-Lucca, Salah Din Mahmud Hasan, Marcia Regina Fagundes-Klen, Maria Luiza Fernandes Rodrigues, Edson Antônio da Silva, Carlos Augusto Rosa, Susana Johann, Cleide Viviane Buzanello

**Affiliations:** 1 Engineering and Exact Sciences Center, State University of West Paraná, Toledo, Paraná Brazil; 2 Department of Microbiology, Federal University of Minas Gerais, Belo Horizonte, Minas Gerais Brazil

**Keywords:** Water quality, Microbial indicators, Environmental yeasts, Antifungal resistance, One Health

## Abstract

Aquatic environments receiving anthropogenic inputs can act as reservoirs for opportunistic microorganisms and antifungal resistance, representing a potential environmental and public health concern. This study evaluated microbial contamination and antifungal susceptibility patterns in surface waters along a rural–urban gradient in a subtropical watershed using an integrated environmental monitoring approach. Physicochemical parameters and conventional microbial (*Escherichia coli*, *Enterococcus* spp., and heterotrophic bacteria) were quantified, and yeast abundance was determined using CHROMagar™ Candida. A total of 327 yeast isolates were evaluated for susceptibility to itraconazole (ITZ) and fluconazole (FCZ) using the CLSI M27-A4 broth microdilution method. Urban sites exhibited markedly higher microbial contamination compared with rural areas, with *Enterococcus* spp. reaching 36,000 colony-forming units (CFU)/100 ml and *E. coli* 19,863 most probable number (MPN)/100 ml. Yeast densities reached up to 351 CFU/100 ml in urban samples, exceeding concentrations typically reported for oligotrophic aquatic systems. Antifungal susceptibility testing revealed a high frequency of resistance among environmental isolates, ITZ (44.3%) compared with FCZ (24.46%). Statistical analyses indicated no significant spatial or seasonal differences in resistance patterns (*p* > 0.05), whereas resistance to ITZ was significantly higher than to FCZ (*p* < 0.001). The results indicate that anthropogenic pressure in urbanized watersheds may contribute to increased microbial contamination and the occurrence of antifungal-resistant yeasts in surface waters. This suggests a potential environmental pathway for the dissemination of resistance traits. The integration of conventional microbial indicators, environmental yeast monitoring, and antifungal susceptibility profiling provides a practical framework for environmental surveillance.

**Graphical Abstract:**

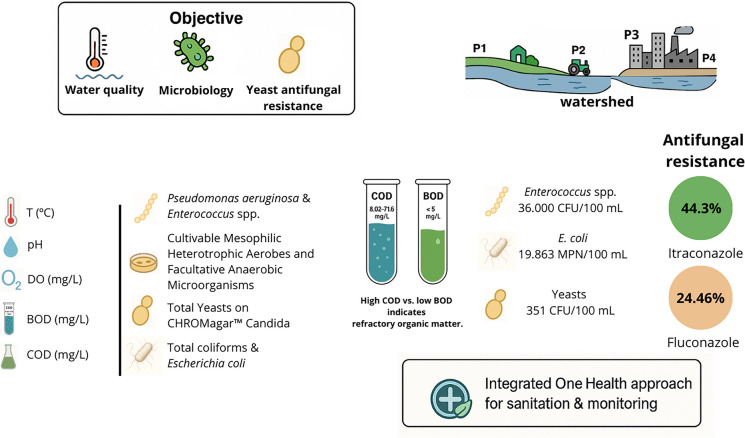

## Introduction

Water resources are essential for both ecological balance and human activities (Raimi et al., 2021). However, water quality has been increasingly compromised by anthropogenic pressures such as deforestation, effluent discharge, sedimentation, and diffuse pollution (Raimi et al., 2021). These factors accelerate eutrophication and alter aquatic ecosystems, with direct consequences for biodiversity and human health (Akhtar et al., 2021). Therefore, monitoring water quality has become a global priority, especially in regions subject to both agricultural and urban pressures.

Conventional assessments rely mainly on physicochemical parameters and fecal indicator bacteria, such as *Escherichia coli* and *Enterococcus* spp. (Bisimwa et al., 2022). While these markers are well established, they may not fully capture the complexity of environmental disturbances (Verma et al., 2025). Yeasts have emerged as complementary bioindicators, since certain species notably *Candida* spp. are associated with environments enriched in organic matter and pollution (Monapathi et al., 2021). According to Cao et al. (2024), the enumeration of yeasts, together with the analysis of heterotrophic microorganisms, provides a broader ecological perspective on surface water contamination.

Another relevant aspect is antifungal resistance, which has become a concern in both clinical and environmental contexts (Endale et al., 2023). Azoles are a class of antifungal compounds widely used in human and veterinary medicine, as well as in agriculture, where they act by inhibiting ergosterol synthesis in fungal cell membranes (European Food Safety Authority et al., ( 2025).

The susceptibility of *Candida* isolates to azoles, such as itraconazole and fluconazole, is therefore of particular interest, as these compounds are among the most commonly used antifungal agents (Rodríguez‐Cerdeira et al., 2025). Changes in susceptibility may reflect adaptive responses to environmental exposure and may have implications for treatment efficacy and public health (Czajka et al., 2023).

Resistance mechanisms, including the overexpression of efflux pumps, can reduce intracellular drug accumulation and are often associated with adaptation to chemically impacted environments, linking environmental contamination with microbial resilience (Lorusso et al., 2022).

The Toledo River (Paraná State, Brazil) exemplifies these challenges, particularly the coexistence of water resource use and anthropogenic pollution. The river extends for 26.5 km and provides water to part of the local population (Manfrin et al., 2018). However, it simultaneously receives domestic, agricultural, and industrial effluents (Manfrin et al., 2018). Differences between rural and urban reaches, together with seasonal variation (rainy summer vs. dry winter), contribute to spatial and temporal heterogeneity in water quality and microbial communities.

In this context, integrative approaches combining physicochemical parameters, conventional microbiological indicators, and environmental yeast monitoring have been increasingly applied to improve the assessment of water quality (Verma et al., 2025). Chromogenic media, such as CHROMagar™, allow the rapid detection and differentiation of yeast groups based on colony characteristics, facilitating ecological interpretation (Monapathi et al., 2021).

In addition, the evaluation of antifungal susceptibility, particularly to widely used azoles such as itraconazole and fluconazole, provides further insight into the occurrence of resistant phenotypes in aquatic environments and their potential implications for environmental and public health (Czajka et al., 2023). Together, these approaches contribute to a more comprehensive understanding of pollution gradients and microbial responses to anthropogenic pressure.

Despite increasing recognition of antimicrobial resistance as a global health threat (World Health Organization, 2025), the environmental dimension of antifungal resistance remains poorly explored, particularly in freshwater systems influenced by both rural and urban activities. Moreover, few studies have integrated conventional microbial indicators with environmental yeast monitoring and antifungal susceptibility profiling to assess pollution gradients in aquatic ecosystems (Stabili et al., 2021; Caicedo-Bejarano et al., 2023; Barros et al., 2024). This gap limits our understanding of how anthropogenic pressures may contribute to the dissemination of antifungal-resistant yeasts in the environment.

This study presents an integrated assessment of aquatic microbial pollution in an urbanizing watershed by combining conventional fecal indicators with environmental yeast monitoring and antifungal susceptibility profiling. The aim was to evaluate whether the integration of these approaches can improve the assessment of aquatic pollution across spatial and seasonal gradients.

Beyond indicating fecal contamination, antifungal susceptibility profiles provide complementary information on the potential impact of anthropogenic activities. Exposure to wastewater, agricultural runoff, and pharmaceutical residues may exert selective pressure on environmental microorganisms, favoring the emergence of azole-tolerant or resistant yeast populations. Therefore, reduced susceptibility to azoles may reflect chronic or cumulative contamination not captured by conventional microbial indicators alone, offering additional insight into pollution sources and environmental health risks.

## Materials and methods

### Study area

The Toledo River watershed is located in the western region of Paraná State, encompassing the municipality of Toledo. Its headwaters arise near São Luiz do Oeste and Gramado Line, and its mouth discharges into the São Francisco Verdadeiro River. The basin covers an area of approximately 26.5 km^2^ and is characterized by a mosaic of rural and urban land.

**Fig. 1:**
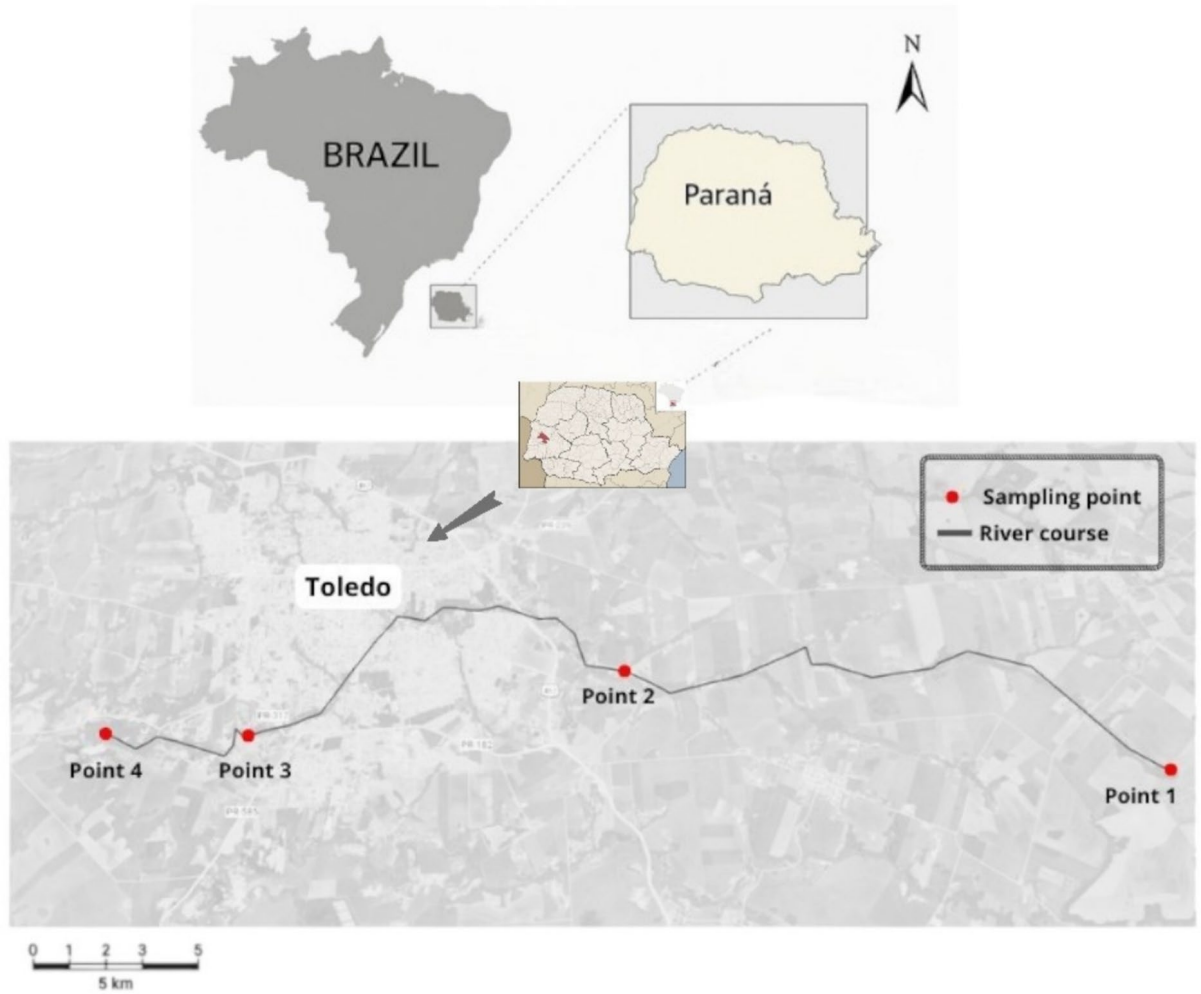
Geographical location of the water collection points in the Toledo River in Paraná

For this study, four sampling points were selected along the river: point 1 (P1) at the headwaters, point 2 (P2) and point 3 (P3) in mid-stream reaches, and point 4 (P4) at the river mouth (Fig. 1). Samples were collected in duplicate, one in March (summer) and one in July (winter).

These points were selected to represent a gradient of land use and anthropogenic pressure, including agriculture, swine farming, urban development, and quarry activity. Table 1 presents the geographic coordinates of each point, the predominant anthropogenic activities, and the classification of reaches as rural or urban (Carvalho et al., 2021).

**Table 1:** Geographic coordinates and anthropogenic activities at the Toledo River (Paraná) sampling points

Sampling point	Geographic coordinates	Anthropogenic activities	Area classification
P1	24° 45′ 45.54″ S and 53° 35′ 02.35″ W	Agriculture/swine Farming	Rural
P2	24° 44′ 50.23″ S and 53° 38′ 19.26″ W	Agriculture	Rural
P3	24° 44′ 10.60″ S and 53° 45′ 06.28″ W	Residential subdivisions/agriculture	Urban
P4	24° 44′ 17.69″ S and 53° 41′ 20.68″ W	Agriculture/swine farming/quarry	Urban

Although the number of sampling points and sampling periods is limited, this design provides an initial exploratory assessment of spatial and seasonal variability along the river, allowing the identification of general patterns associated with land use and anthropogenic influence. The results should therefore be interpreted as indicative trends rather than definitive conclusions.

Water samples were collected in 500-ml high-density polyethylene bottles, previously rinsed with site water. Collection of the water was performed at mid-depth locations at least 1 m from the bank, during March and July. Immediately after sampling, bottles were stored in insulated coolers with ice and transported to the laboratory. At the laboratory, water samples were processed within 6–8 h after collection to preserve microbial integrity, following standard recommendations for environmental microbiological analyses (APHA, 2017; ISO 19458, 2006). Samples were filtered through cellulose ester membranes (0.45 µm and 0.22 µm pore sizes) for subsequent microbiological and physicochemical analyses.

### Physicochemical parameters

Physicochemical parameters (temperature, pH, and dissolved oxygen (DO)) were measured in situ using a portable multiparameter probe (AAKER), followed by biochemical oxygen demand (BOD) and chemical oxygen demand (COD) analyses (APHA, 2012). COD was determined by the closed-reflux method 5220 D, and BOD was measured according to method 5210 B, with a 5-day incubation at 20 °C. All assays were conducted in duplicate to evaluate organic load and the capacity of the aquatic environment to degrade organic matter, parameters commonly used to assess ecosystem functioning and anthropogenic pressure in freshwater systems (APHA, 2017; Chapman, 2021).

### Microbiological parameters

To characterize and quantify the microbiological profile at four sampling points along the Toledo River, specific microbiological analyses were conducted in triplicate. These analyses included the enumeration of different groups of water-quality indicator microorganisms, enabling the assessment of biological contamination at each study point.

**Fig. 2:**
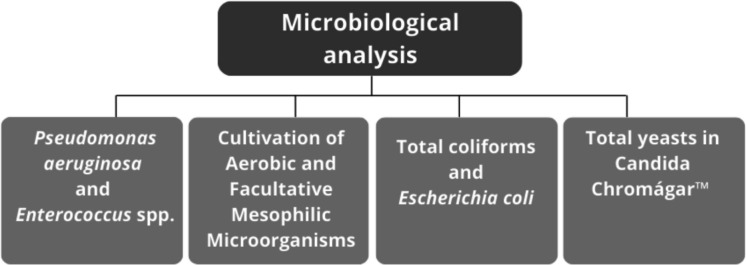
Schematic representation of the procedures used to determine the microbiological profile of the four Toledo River (Paraná) sampling points

For membrane filtration-based analyses, independent aliquots of each sample were filtered separately for each target microorganism (e.g., *Pseudomonas aeruginosa* and *Enterococcus* spp.), using selective culture media according to standard methods. In contrast, heterotrophic bacteria were quantified using the pour-plate technique from the same set of serial dilutions prepared for each sample. Figure 2 schematically illustrates the procedures followed for the microbiological analyses at these locations.

#### Enumeration of *Pseudomonas aeruginosa* and *Enterococcus* spp.

For the quantification of *Pseudomonas aeruginosa* and *Enterococcus* spp., 100 ml of each water sample was filtered through cellulose ester membranes (0.45 µm pore size) (APHA, 1995). *P. aeruginosa* was detected using method 9213 E (membrane filtration), with membranes placed on selective cetrimide agar and incubated at 35–37 °C for 24–48 h. Typical colonies were identified based on characteristic pigmentation and fluorescence under UV light.

*Enterococcus* spp. were enumerated using method 9230 C, with membranes placed on m-Enterococcus agar and incubated at 35 °C for 48 h. Colonies presenting typical morphology were counted as presumptive enterococci. These indicators were used to evaluate water quality and identify potential fecal contamination sources, as widely recommended for microbial assessment of aquatic environments (APHA, 2017).

#### Enumeration of cultivable mesophilic heterotrophic aerobes and facultative anaerobic microorganisms

Quantitative analysis of total cultivable mesophilic heterotrophic aerobes and facultative anaerobes was performed using 1 ml aliquots of each water sample. Undiluted samples and serial dilutions (10^−1^ to 10^−4^ in 0.15 M saline) were plated using the pour-plate technique. Standard plate count agar was prepared and sterilized according to the manufacturer’s instructions and poured over the inoculated samples to ensure homogeneous distribution. Plates were incubated at 35 °C for 24 h (Mani-López et al., 2022).

After incubation, colony-forming units (CFU) were counted using a colony counter. Results were expressed as CFU/100 ml and used to assess microbial load and spatial variations in water quality among sampling sites. Results were expressed as CFU/100 ml and used as an indicator of overall microbial load, supporting the evaluation of water quality and anthropogenic influence across sampling sites.

#### Determination of total coliforms and *Escherichia coli* by the most probable number method

Analyses for total coliforms and *E. coli* were conducted following APHA (2012), specifically method 9223-B, which employs an enzyme–substrate coliform test. A commercial Colilert-18® kit was used to detect and quantify total coliforms and *E. coli* simultaneously. This kit relied on a substrate technology containing specific enzymes: following sample inoculation into the medium, incubations were carried out at 35 °C for 24 h. During this period, enzymatic reactions produced a color change in the medium indicative of coliform presence (Manzanas et al., 2023). Results were expressed as the most probable number (MPN) per 100 ml, calculated via standard probability tables to estimate microorganism density in each sample.

#### Enumeration of total yeasts on CHROMAGAR™ Candida

Yeast counts were conducted in quintuplicate to ensure reliability, following APHA (2012) protocols. Three replicates of water samples were filtered through cellulose-ester membranes (0.45 µm and 0.22 µm pore sizes) using sample volumes of 1 ml, 5 ml, and 10 ml. The remaining two replicates (100 µl and 200 µl) were processed by the spread-plate technique. Each membrane was placed onto CHROMagar™ Candida plates (47.7 g/l), which differentiate *Candida* species by colony color. Plates were incubated at 35 °C, with readings at 24 h, 48 h, and 72 h, and results were recorded as CFU/100 ml. Isolates were subsequently subcultured on CHROMagar™ Candida by streak plate to identify species: each *Candida* species expresses a specific enzyme that cleaves a unique chromogenic substrate, producing characteristic colony colors for visual differentiation (Perry & Freydière, 2007).

Yeasts were cultured on CHROMagar™ Candida for presumptive identification, allowing differentiation into yeast groups or species complexes based on colony color and morphology (Odds & Bernaerts, 1994). As this medium does not provide definitive taxonomic resolution, all isolates were consistently described as presumptively identified yeast groups throughout the study. This approach is widely used for rapid environmental screening of *Candida*-like yeasts.

### Antifungal susceptibility testing

The susceptibility of yeast isolates to antifungal agents was evaluated by the broth microdilution method following Clinical and Laboratory Standards Institute guidelines (CLSI M27-A4) for determination of minimum inhibitory concentration (MIC) against itraconazole (ITZ) and fluconazole (FCZ). Colony morphology was used as a preliminary criterion to select representative isolates for testing (Chantratita et al., 2007). Colonies were differentiated based on macroscopic characteristics, including color, size, texture, and margin (Kurtzman et al., 2011), as observed on CHROMagar Candida plates. Isolates displaying distinct morphotypes were selected to avoid redundancy and ensure representation of the phenotypic diversity present in the samples.

#### Preparation of antifungal agents

Stock solutions (1 mg/ml) of ITZ and FCZ were prepared. ITZ was dissolved in dimethyl sulfoxide and stored at − 20 °C; FCZ was dissolved in sterile distilled water and stored under the same conditions. Eleven twofold serial dilutions of each antifungal were tested (Table 2). Negative controls contained only RPMI medium, without the inoculum.

**Table 2:** Antifungal concentrations (µg/ml) tested for MIC determination

Antifungal	Concentrations (µg/ml)									
Fluconazole	64	32	16	8	4	2	1	0.5	0.25	0.125
Itraconazole	16	8	4	2	1	0.5	0.25	0.125	0.06	0.03

#### Inoculum preparation

Yeasts were grown on Sabouraud dextrose agar for 24 h at 35 °C. Resulting colonies were suspended in 5 ml of 1.5 M saline. Cell density was adjusted spectrophotometrically to 75–77% transmittance at 530 nm, corresponding to ∼0.5 × 10^6^ CFU/100 ml. Two additional serial dilutions (1:50 and 1:20) were performed to achieve a final inoculum of ∼0.5 × 10^3^ CFU/100 ml. Aliquots of yeast suspension (100 µl) were dispensed into the wells of flat-bottom 96-well microtiter plates containing 100 µl of each antifungal dilution (Garrigues et al., 2018). Plates were incubated at 35 °C. Visual readings were taken after 24 h and 48 h.

#### Determination of MIC

MIC was defined as 100% growth inhibition for ITZ and 50% inhibition for FCZ. Results were interpreted according to the breakpoints established by CLSI M27-A4 (de Sousa et al., 2020). For FCZ, breakpoints were susceptible (S) (≤ 8 µg/ml), dose-dependent (DD) (16–32 µg/ml), and resistant (R) (≥ 64 µg/ml). For ITZ, breakpoints were susceptible (≤ 0.125 µg/ml), dose-dependent (0.25–0.5 µg/ml), and resistant (≥ 1 µg/ml). These criteria were used to classify each isolate’s susceptibility to the antifungals tested.

#### Calculation of MIC_50_ and MIC_90_

MIC_50_ and MIC_90_ were calculated as the lowest concentrations of each antifungal required to inhibit 50% and 90% of the isolates, respectively.

The study design was intended as an environmental surveillance case study, focusing on spatial and seasonal patterns, rather than causal inference.

### Statistical analysis

Microbial data were log_10_ transformed to reduce skewness and improve normality, as commonly recommended for microbial count data (Sokal & Rohlf, 1987; Zar, 1999). Due to the small sample size and non-normal distribution, non-parametric tests were applied. Differences between rural and urban sites were assessed using the Mann–Whitney *U* test, a robust method for comparing independent groups without assuming normality (Conover, 1999). Exact *p* values were calculated, and a significance level of *p* < 0.05 was adopted.

Antifungal susceptibility data were analyzed using contingency tables, with outcomes dichotomized as R and non-resistant (NR; S + susceptible dose-dependent (SDD)). Fisher’s exact test (two-sided) was used due to small, expected frequencies. Effect sizes were expressed as odds ratios (OR) with 95% confidence intervals (CI).

Principal component analysis (PCA) was performed on standardized variables (*z*-scores) to explore relationships among physicochemical and microbiological parameters. All statistical analyses were conducted using Statistica software (version 14.0).

## Results and discussion

### Physicochemical analyses

The physicochemical quality of the Toledo River water was assessed by measuring temperature, pH, DO, BOD, and COD (Table 3). Reported values represent the mean for the winter and summer sampling periods. These parameters are essential for understanding ecosystem functioning and for evaluating compliance with the CONAMA Resolution 357/2005, which establishes water quality standards for public supply, recreation, and the protection of aquatic life.

**Table 3:** Physicochemical parameters of Toledo River water in winter and summer, compared to CONAMA 357/2005 class 2 limits

Parameter	Rural P1	Rural P2	Urban P3	Urban P4	CONAMA 357/2005 (class 2)
Temperature (°C)	18.5 (W)/20.6 (S)	17.9 (W)/20.6 (S)	18.0 (W)/ 22.3 (S)	17.5 (W)/ 22.9 (S)	≤ 40 °C (variation ≤ 3 °C)
pH	6.43 (W)/6.92 (S)	6.39 (W)/6.88 (S)	6.34 (W)/6.94 (S)	6.35 (W)/7.00 (S)	6.0–9.0
DO (mg/l)	5.10 (W)/5.79 (S)	5.92 (W)/5.48 (S)	7.22 (W)/7.69 (S)	5.30 (W)/5.42 (S)	≥ 5.0 mg/l
BOD(mg/l)	< 1.00 (W)/< 1.00 (S)	< 1.00 (W)/< 1.00 (S)	2.51 (W)/4.26 (S)	1.77 (W)/3.32 (S)	≥ 5.0 mg/l
COD(mg/l)	51.67 (W)/15.21 (S)	71.67 (W)/8.02 (S)	71.67 (W)/34.80 (S)	64.67 (W)/38.50 (S)	No defined limit

*W* winter, *S* summer

Overall, all parameters complied with CONAMA 357/2005 class 2 regulatory limits; however, spatial and seasonal variations indicate the influence of environmental and anthropogenic factors. Temperature values increased during summer, particularly at urban site P4, likely due to local anthropogenic contributions combined with higher ambient temperatures, as reported by Gorde and Jadhav (2013). Elevated temperatures are known to reduce oxygen solubility and affect aquatic biodiversity (Anh et al., 2023), while also accelerating microbial and chemical processes (Bonacina et al., 2023; Amini Tabrizi et al., 2023).

pH values remained within the range of 6.3 to 7.0 across all sites, suggesting stable conditions and buffering capacity, which supports biogeochemical processes and aquatic life (Mullungal et al., 2024; Pinheiro et al., 2021). Dissolved oxygen levels consistently exceeded the minimum threshold (5.0 mg/l) required by legislation CONAMA 357/2005. However, lower values were observed during summer, likely associated with increased temperature and organic load, which enhance microbial respiration and oxygen consumption (Chapra et al., 2021; McCabe et al., 2021). These seasonal variations suggest that even when regulatory thresholds are met, aquatic organisms may experience periods of environmental stress.

BOD values remained below 5.0 mg/l at all sites, indicating relatively low levels of biodegradable organic matter. In contrast, COD values were higher in urban areas, particularly during winter, suggesting the presence of recalcitrant organic compounds. The discrepancy between COD and BOD has been associated with non-biodegradable pollutants such as synthetic compounds, agro-industrial residues, and pesticides (Kumar et al., 2021; Kumari & Kumar, 2023; Liu et al., 2022; Lv et al., 2024).

Seasonal rainfall also influenced water quality dynamics, particularly variations in organic load (BOD and COD), DO, and microbial abundance. Rainfall contributed to dilution effects during summer while also increasing pollutant input through runoff (INMET, 2023; SIMEPAR, 2023; Wei et al., 2023). Findings from the present study highlight that conventional physicochemical indicators alone may underestimate environmental risks, particularly when refractory organic matter predominates.

The limited number of sampling points and sampling campaigns represents a constraint of the present study. Therefore, the observed spatial and seasonal patterns should be interpreted with caution and considered as preliminary evidence.

### Microbiological analyses

#### Fecal indicators and opportunistic pathogens

Microbial indicators showed clear spatial and seasonal variation (Table 4), with higher counts observed during summer, consistent with seasonal patterns reported in aquatic systems (Storto et al., 2021).

**Table 4:** Seasonal enumeration of *P. aeruginosa* and *Enterococcus* spp. in Toledo River water (CFU/100 ml)

**Microorganism** **(CFU/100 ml)**	**Rural**				**Urban**			
	**P1**		**P2**		**P3**		**P4**	
	**Winter**	**Summer**	**Winter**	**Summer**	**Winter**	**Summer**	**Winter**	**Summer**
*Pseudomonas aeruginosa*	< 1	< 1	< 1	100	< 1	< 1	< 1	400
*Enterococcus* spp.	13.000	15.000	2.000	18.000	12.000	18.000	19.000	36.000

*Enterococcus* spp. counts ranged from 2.000 to 36.000 CFU/100 ml, exceeding international standards for recreational water quality, including those established by the US Environmental Protection Agency (Epa, 2012) and the European Directive 2006/7/EC. *Enterococcus* spp. are fecal indicator bacteria that originate from the gastrointestinal tract of mammals (Li et al., 2021). Their high abundance, particularly in urban sites, strongly suggests contamination by untreated domestic wastewater.

*Pseudomonas aeruginosa* was below detection limits in most samples, according to the sensitivity of the membrane filtration method (APHA, 1995), but reached 100 CFU/100 ml at P2 and 400 CFU/100 ml at P4 during summer. Although not regulated in Brazilian legislation, its presence is relevant due to its pathogenic potential and association with organic pollution and wastewater contamination (Męcik et al., 2024; Wood et al., 2023). These findings indicate that microbial contamination increases along the river, particularly in urbanized areas, reflecting cumulative anthropogenic inputs. Similar trends have been reported in rivers influenced by urbanization, where fecal indicators increase downstream (Sharma et al., 2024).

#### Heterotrophic bacteria

Heterotrophic microorganisms showed a marked increase from rural to urban sites (Table 5), reflecting the influence of organic matter availability on microbial growth (Madigan et al., 1997). In rural areas, counts ranged from 7.7 × 10^3^ to 3.5 × 10^4^ CFU/100 ml, whereas in urban areas, values reached up to 6.1 × 10^5^ CFU/100 ml during summer.

**Table 5:** Counts of cultivable mesophilic heterotrophic aerobes and facultative anaerobes in Toledo River water

Microorganism (10^4^ CFU/100 ml)	Rural				Urban			
	**P1(W)**	**P1(S)**	**P2(W)**	**P2(S)**	**P3(W)**	**P3(S)**	**P4(W)**	**P4(S)**
Heterotrophs	1.01	2.08	0.77	3.5	21.9	36.1	47.2	61.0

Seasonal variation was also observed, with higher counts during summer across all sites. This pattern is consistent with increased runoff transporting organic matter and nutrients, combined with elevated temperatures that stimulate microbial metabolism (Taborda et al., 2022; Zhang et al., 2022). The co-occurrence of high heterotrophic counts with elevated *Enterococcus* spp. and *P. aeruginosa* further indicates chronic wastewater contamination, particularly in urban reaches.

#### Total coliforms and *Escherichia coli*

The distribution of total coliforms and *E. coli* (Table 6) further confirmed the influence of anthropogenic activities. As shown in Table 6, only site P2 complied with the limits established by the CONAMA Resolution 357/2005, while all other sites exceeded the acceptable thresholds.

**Table 6:** Counts of total coliforms and *E. coli* in Toledo River water

Microorganism (CFU/100 ml)	Rural reach				Urban reach			
	**P1(W)**	**P1(S)**	**P2(W)**	**P2(S)**	**P3(W)**	**P3(S)**	**P4(W)**	**P4(S)**
Total coliforms	3.436	5.475	4.106	9.208	9.606	24.196	24.196	24.196
*E. coli*	633	1.187	480	723	1.334	3.873	9.208	19.863

*E. coli* concentrations were consistently higher during summer, particularly at downstream site P4, suggesting pollutant accumulation along the river continuum. These findings are consistent with increased runoff and higher temperatures that favor bacterial proliferation (Bhatt et al., 2024).

Regulatory thresholds for *E. coli* vary across the regions. The US EPA recommends a geometric mean of 126 CFU/100 ml, while European standards classify waters as “excellent” at ≤ 250 CFU/100 ml. In contrast, Brazilian legislation (CONAMA, 2000) is more permissive. Regardless of the framework applied, most sites in the Toledo River exceeded acceptable limits, consistent with compromised water quality. As *E. coli* is a recognized fecal indicator organism (Makuwa et al., 2020), these results strongly suggest contamination by human or animal waste, particularly in urban areas.

#### Statistical analysis of spatial differences

To quantitatively assess spatial differences, microbial data were log transformed and analyzed using the Mann–Whitney *U* test. Significant differences were observed between rural and urban sites for both log(*E. coli*) and log(heterotrophic) (Table 7), with higher values in urban areas. Considering the small sample size (*n* = 4 per group), exact *p* values were also evaluated, confirming statistical significance.

**Table 7:** Mann–Whitney test results

Variable	*U*	*Z*	*p* value	Exact *p*
log(*E. coli*)	0.00	− 2.17	0.030	0.028
log(Heterotrophic)	0.00	− 2.17	0.030	0.028

**Fig. 3:**
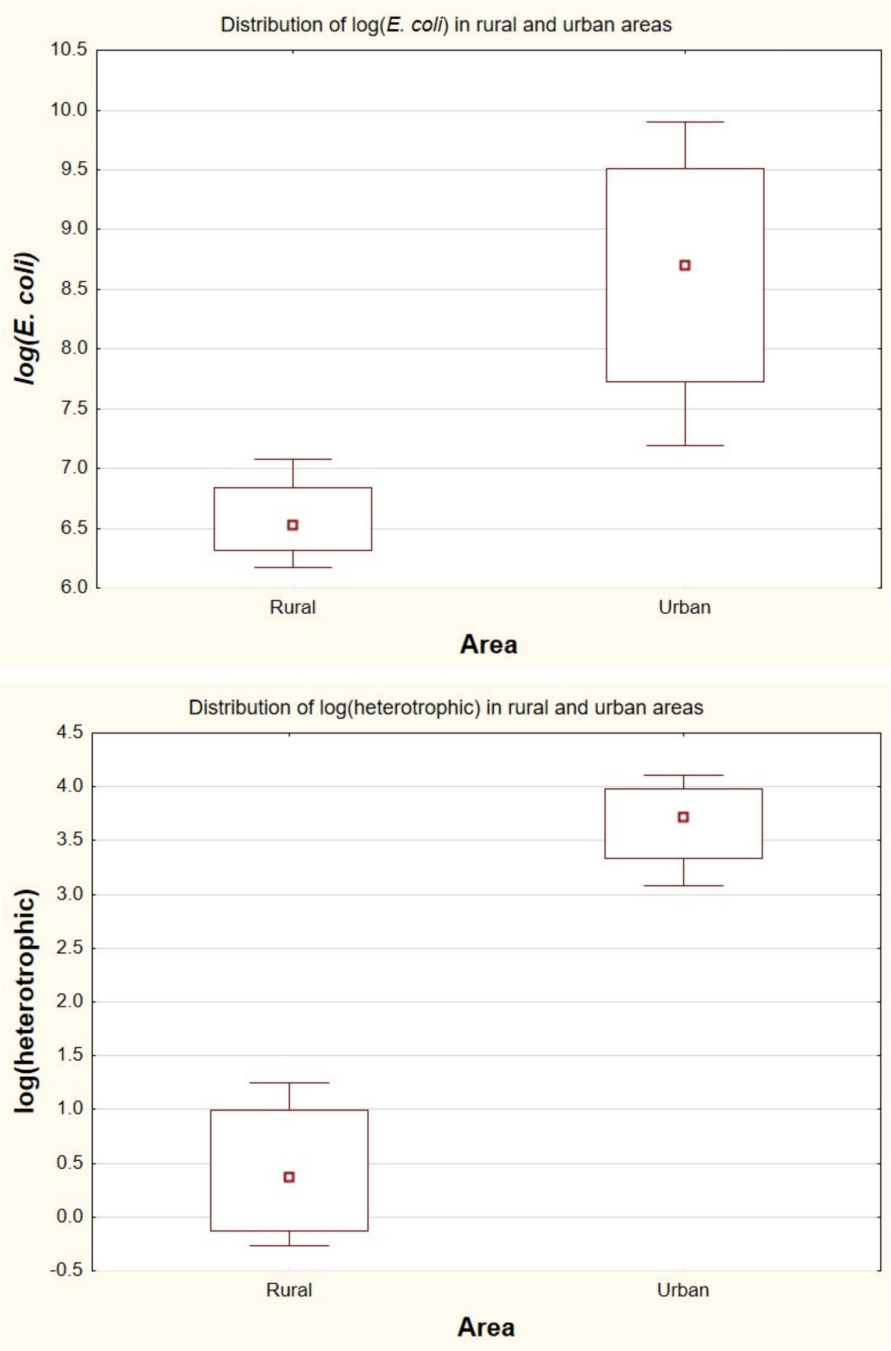
Boxplots showing the distribution of log-transformed *E. coli* and heterotrophic bacteria counts in rural and urban sites

Boxplot analysis (Fig. 3) showed higher median values and greater variability at urban sites. This pattern suggests increased microbial contamination driven by anthropogenic inputs.

#### Multivariate analysis (PCA)

**Fig. 4:**
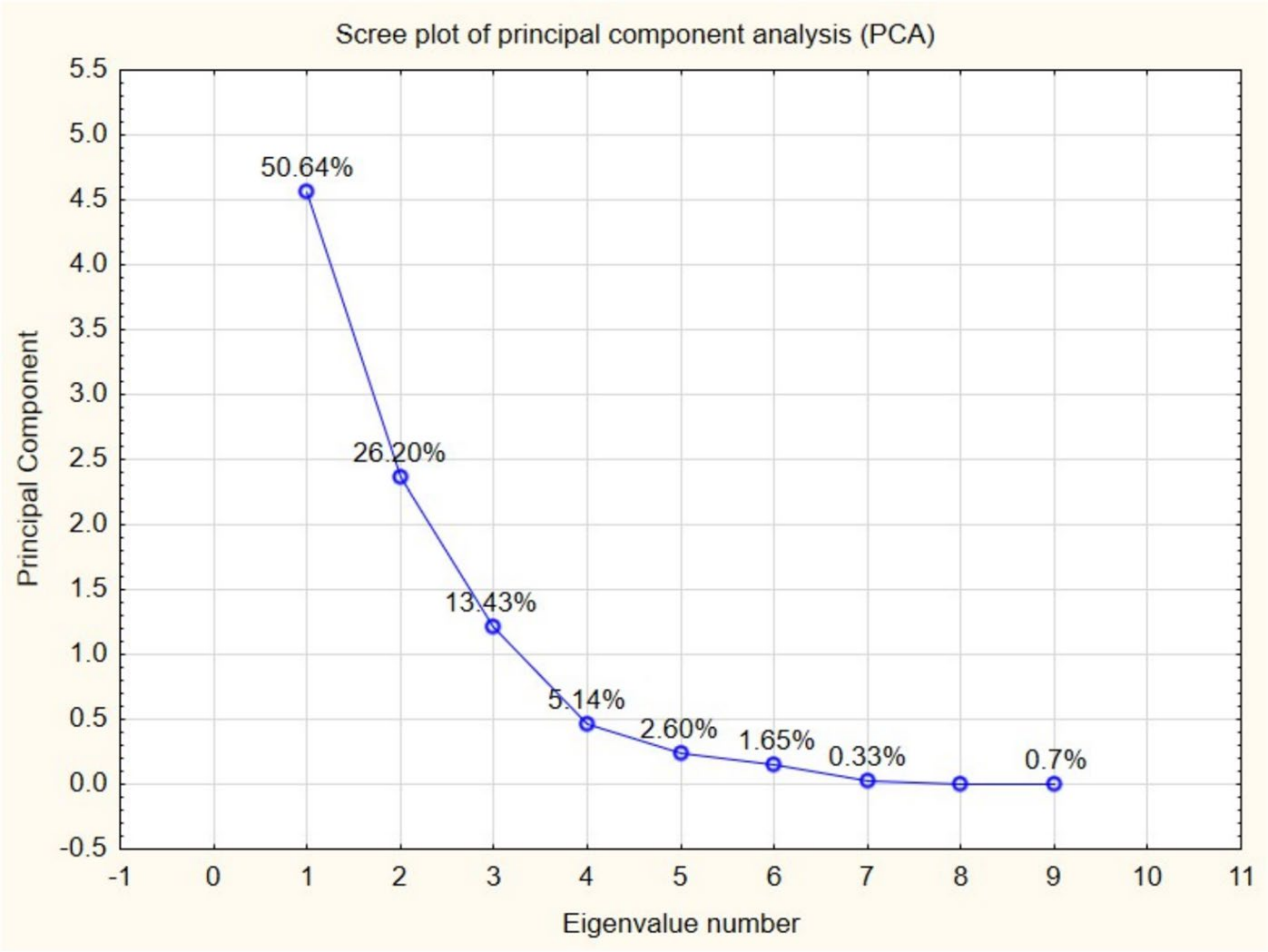
Scree plot of PCA showing the proportion of variance explained by each component. The first two principal components accounted for 76.84% of the total variance

PCA was applied to explore relationships among microbial and physicochemical variables. The scree plot (Fig. 4) shows that the first principal component (PC1) explained 50.64% of the total variance, while the second principal component (PC2) explained 26.20%, resulting in a cumulative variance of 76.84%. This indicates that most of the variability in the dataset is captured by the first two components.

**Fig. 5:**
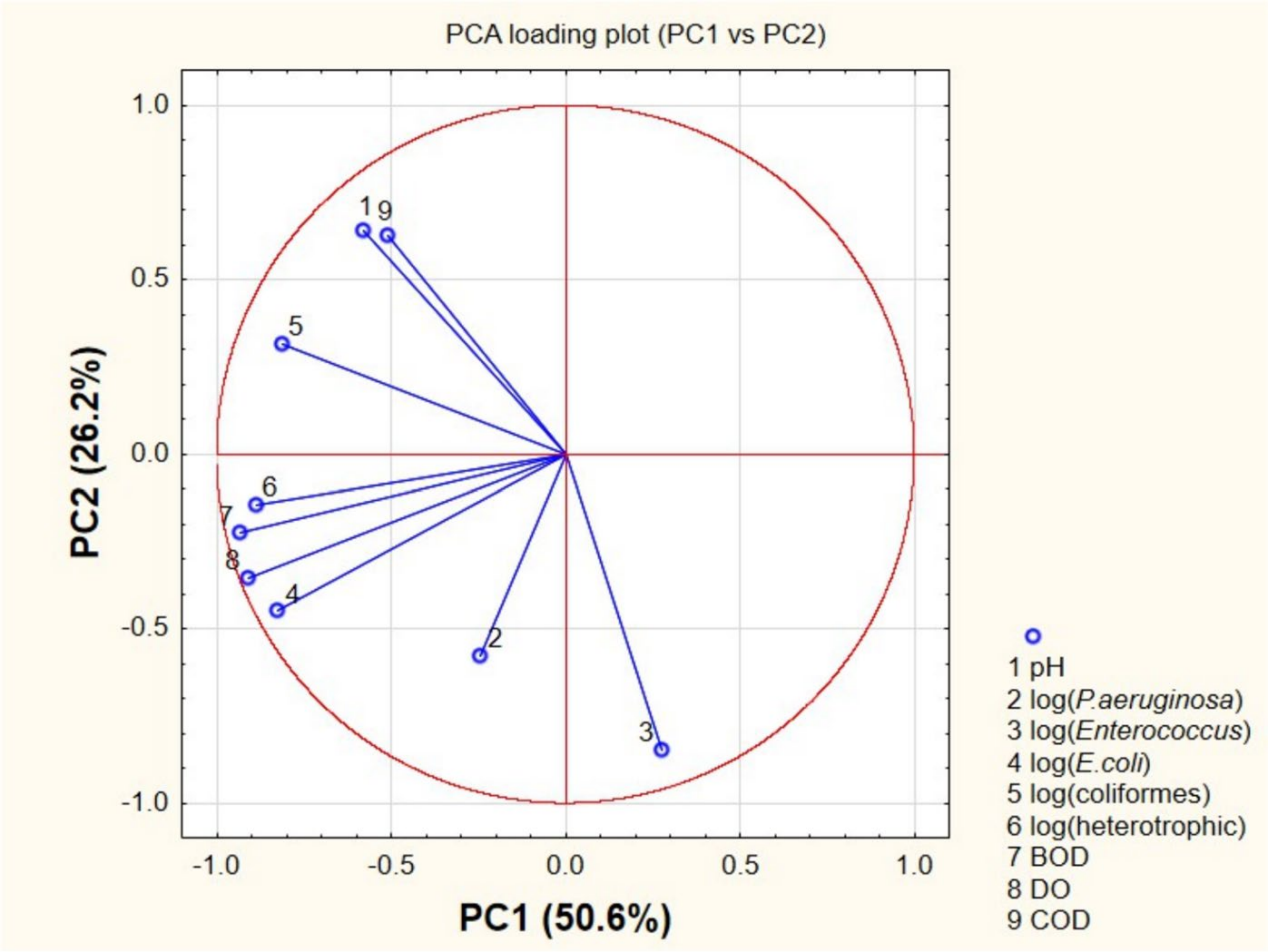
PCA loading plot illustrating relationships among physicochemical and microbiological variables. Variables with similar orientations are positively correlated, whereas opposite directions indicate negative correlations

**Fig. 6:**
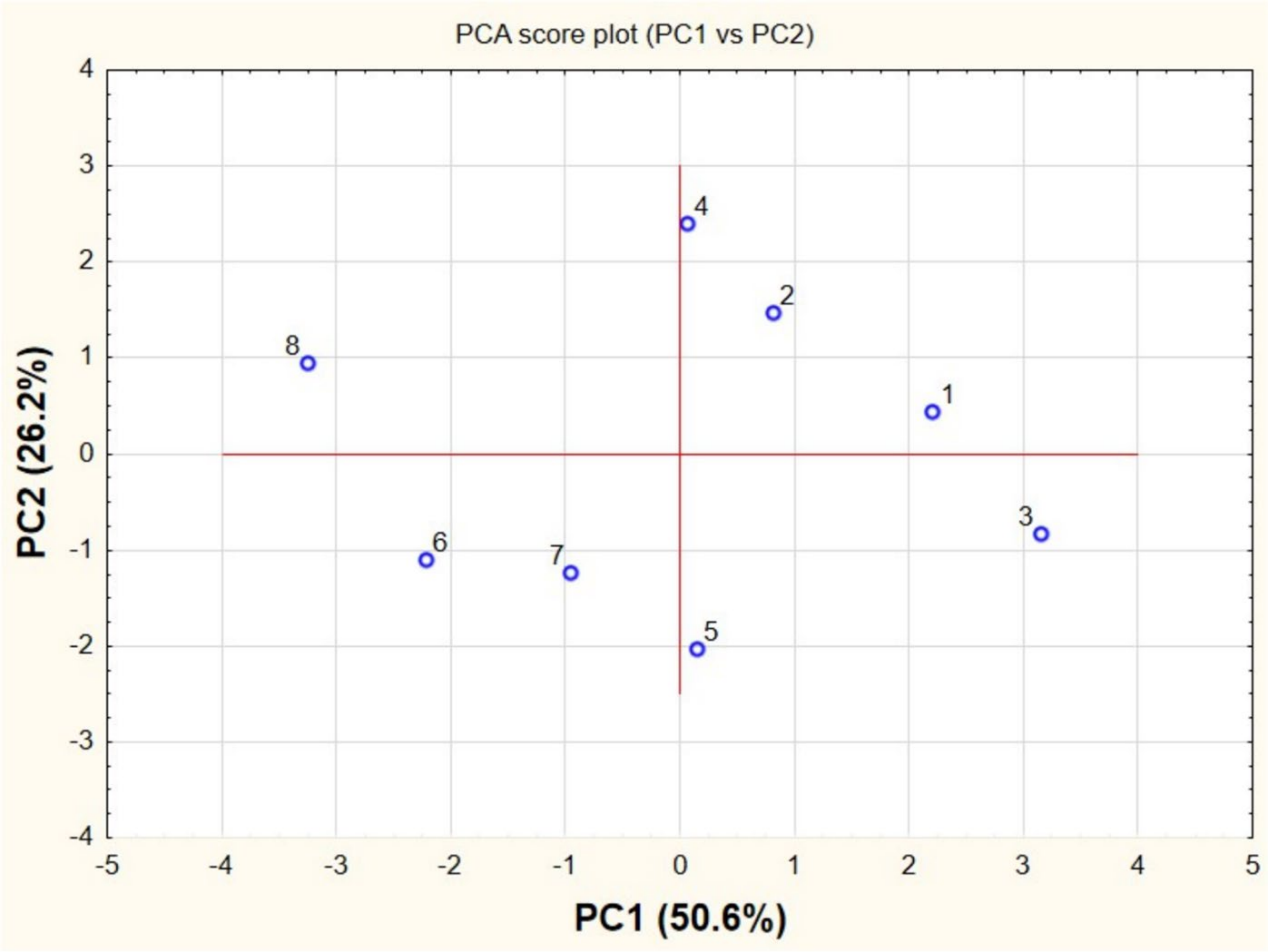
PCA score plot showing sample distribution along the first two principal components. Clustering patterns reflect differences between rural and urban sites

In the score plot (Fig. 5), urban sampling sites were associated with higher values of microbial indicators, while rural sites showed lower values. The loading plot (Fig. 6) revealed that *E. coli*, total coliforms, *Enterococcus* spp., and heterotrophic bacteria were oriented in the same direction, indicating positive correlations among these variables. This pattern suggests a common contamination source, typically associated with fecal inputs (Saied et al., 2025).

These variables were also associated with organic matter indicators (COD and BOD), indicating that microbial proliferation is linked to organic pollution. In contrast, dissolved oxygen showed an inverse relationship with these parameters, reflecting oxygen consumption during microbial degradation processes (Cheng et al., 2024). The score plot (Fig. 6) showed the spatial distribution of samples along environmental gradients, reflecting differences in contamination levels between sites.

The PCA results further supported the separation between rural and urban sites, indicating that microbial indicators and physicochemical parameters were associated with anthropogenic influence. These multivariate patterns reinforce the interpretation that urbanization plays a key role in shaping microbial dynamics in the studied watershed (Helena et al., 2000). This multivariate approach reduced data complexity and allowed the identification of underlying patterns associated with anthropogenic pressure (Jolliffe, 2025).

#### Integrated interpretation and conceptual model

The integration of physicochemical, microbiological, and statistical analyses supports a conceptual model in which urbanization drives water quality degradation. Anthropogenic inputs, including untreated wastewater and surface runoff, increase organic matter and microbial contamination, promoting microbial growth and oxygen consumption (Khatri & Tyagi, 2015). This process leads to reduced dissolved oxygen levels and overall deterioration of water quality.

These patterns are consistent with the “urban stream syndrome,” where urbanization leads to increased pollutant loads and altered ecosystem functioning (MacKenzie et al., 2022). From a One Health perspective, elevated microbial loads may increase the risk of pathogen dissemination and environmental antimicrobial resistance (Endale et al., 2023), highlighting the importance of integrated monitoring strategies.

The agreement between descriptive data, statistical analysis, and multivariate approaches strengthens the findings and demonstrates that microbial contamination and organic pollution are interconnected processes driven by anthropogenic pressure.

#### Total yeasts on CHROMagar™ Candida

Yeast isolates were selected based on their high probability of belonging to the genus *Candida*, given its relevance as an environmental bioindicator (Caicedo-Bejarano et al., 2023). Yeast density was expressed as CFU/100 ml of water sample. In rural sites, mean concentrations were 28 CFU/100 ml in summer and 35 CFU/100 ml in winter. In urban sites, densities reached 79 CFU/100 ml in summer and 351 CFU/100 ml in winter. According to Novak Babič et al. (2017), oligotrophic and well-preserved environments typically exhibit yeast concentrations below 100 CFU/100 ml.

Seasonal variation was limited to rural sites (28 CFU/100 ml vs. 35 CFU/100 ml). Urban sites showed a more pronounced increase in winter, with yeast densities rising more than fourfold. This pattern suggests that anthropogenic inputs may exert a stronger influence than seasonal variability. Urban site values exceeded oligotrophic threshold by approximately fivefold. This supported the interpretation that urbanized sections are associated with higher organic matter loads and greater eutrophication.

Recent taxonomic revisions have reassigned several species previously classified within the genus *Candida* to other genera (e.g., *Nakaseomyces glabrata*, *Pichia kudriavzevii*). However, isolates were reported here as *Candida* spp. to maintain consistency with CHROMagar™ Candida classification and with previous environmental studies (Ozcan et al., 2010).

For preliminary identification, CHROMagar™ Candida was employed due to its ability to differentiate species based on colony color and morphology (Odds & Bernaerts, 1994). This method has been validated in both clinical and environmental studies, demonstrating high agreement with confirmatory techniques such as matrix-assisted laser desorption/ionization time-of-flight mass spectrometry (MALDI-TOF MS). For instance, Taverna et al. (2023) reported that 94% of isolates were correctly identified at the species or complex level using CHROMagar™, later confirmed by MALDI-TOF analysis, supporting its reliability as a screening approach.

Nevertheless, CHROMagar™ Candida does not provide definitive taxonomic identification. Molecular or proteomic techniques such as ribosomal DNA (rDNA) sequencing or MALDI-TOF MS remain necessary for species-level confirmation. Despite this limitation, CHROMagar™ represents a practical and validated tool for preliminary identification in environmental monitoring studies.

**Fig. 7:**
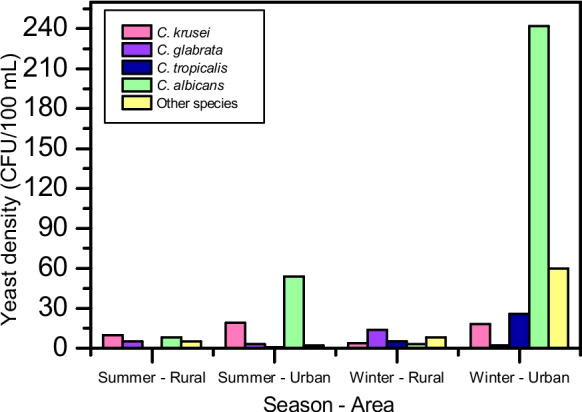
Spatial and seasonal distribution of *Candida* spp. in rural (P1, P2) and urban (P3, P4) sites during summer and winter. Values represent yeast density (CFU/100 ml)

Figure 7 illustrates the spatial and seasonal distribution of *Candida* spp. based on CFU counts. Yeast densities were consistently higher in urban sites, particularly during winter. In summer, *Candida krusei* (10 CFU/100 ml) and *Candida albicans* (8 CFU/100 ml) predominated in rural sites, whereas in urban sites, *C. albicans* (54 CFU/100 ml) and *C. krusei* (19 CFU/100 ml) were more abundant. During winter, this contrast became more pronounced, with *C. albicans* reaching 242 CFU/100 ml in urban sites while remaining low in rural sites (3 CFU/100 ml). Additionally, winter samples from urban sites showed an increase in other species (60 CFU/100 ml), suggesting a broader diversification of the yeast community under conditions of higher organic load.

The higher abundance of presumptive *Candida albicans*-like colonies in urban environments may be associated with the discharge of domestic and industrial effluents, as this group is commonly linked to organic-rich environments and anthropogenic contamination (Caicedo-Bejarano et al., 2023).

Less abundant but ecologically relevant yeast groups, including colonies presumptively identified as *Candida tropicalis*- and *Candida glabrata*-like, were also more frequent in urban sites during winter. These patterns support the interpretation that both urbanization and seasonal conditions influence yeast community structure.

Previous studies have demonstrated that pristine aquatic environments typically exhibit lower yeast densities than eutrophic systems (Monapathi et al., 2017). Kumar et al. (2024) reported a strong correlation between yeast abundance and other microbiological indicators, including fecal coliforms, total coliforms, and *E. coli*. Opportunistic yeasts, particularly those of the genus *Candida*, can persist and proliferate in nutrient-rich environments, making them useful indicators of organic pollution (Monapathi et al., 2020).

Furthermore, several studies (Celekli & Şahin, 2021; Sagova-Mareckova et al., 2021; Choix et al., 2023) have emphasized the ecological value of microbial bioindicators in assessing the impact of industrial, agricultural, and domestic effluents. In this context, *Candida* species may serve as complementary indicators of water quality. Their ability to persist in contaminated environments, often longer than traditional fecal indicators, makes them useful for detecting chronic anthropogenic pollution (Samson et al., 2020).

The co-occurrence of elevated yeast densities and increased levels of conventional microbial indicators in urban sites suggests that environmental yeasts may serve as complementary bioindicators of organic pollution. Unlike traditional fecal indicators, yeasts can persist and proliferate under nutrient-rich conditions, reflecting chronic contamination and ecosystem disturbance (Naranjo‐Ortiz & Gabaldón, 2019). This combined approach may enhance the sensitivity of water quality assessments, particularly in environments impacted by diffuse pollution sources.

### Antifungal susceptibility testing

The MIC represents the lowest antifungal concentration required to inhibit visible fungal growth. In this study, 327 yeast isolates were selected from colonies grown on CHROMagar™ Candida to represent the phenotypic diversity observed across sampling sites and seasons. Selection was based on colony color, size, texture, margin, and morphology (Vanden Bossche et al., 1994). When multiple colonies with the same morphotype were observed on a plate, representative colonies were selected to avoid redundant testing of visually identical isolates. Thus, the susceptibility dataset represents presumptively identified yeast morphotypes recovered from rural and urban reaches during summer and winter. Susceptibility to itraconazole (ITZ) and fluconazole (FCZ) was evaluated according to CLSI M27-A4 guidelines.

**Fig. 8:**
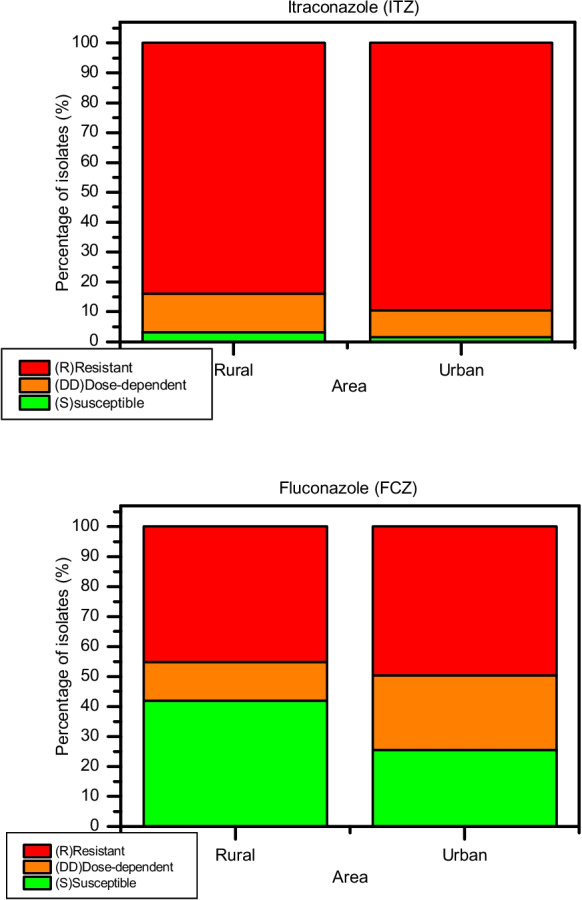
Distribution of antifungal susceptibility profiles of yeast isolates from rural and urban reaches of the Toledo River. Stacked bars represent the percentage of isolates classified as resistant (R), susceptible dose-dependent (SDD), and susceptible (S) for ITZ and FCZ

The distribution of susceptibility categories across sampling areas is illustrated in Fig. 8. A predominance of resistant isolates was observed for ITZ in both rural and urban reaches, whereas FCZ exhibited a more heterogeneous distribution, with higher proportions of S and DD isolates.

Antifungal resistance in yeasts arises from multiple mechanisms, including reduced drug–target affinity, overexpression of target enzymes, and enhanced activity of efflux pumps (Gow et al., 2022). This leads to decreased intracellular drug accumulation, and impaired drug uptake. In contrast, antifungal tolerance represents a reversible physiological adaptation in which a subpopulation of cells persists under inhibitory drug concentrations, often mediated by stress-response pathways (Lee et al., 2023). Susceptibility classifications included R, DD, and S, following standard antifungal susceptibility testing frameworks.

Overall, susceptibility testing revealed a predominance of resistance to ITZ. Among all isolates, 145 (44.3%) were classified as R, 16 (4.89%) as DD, and only 3 (0.91%) as S. In contrast, FCZ showed lower resistance frequencies, with 80 isolates (24.46%) classified as R, 37 (11.31%) as DD, and 47 (14.37%) as S. These findings are consistent with Caicedo-Bejarano et al. (2023) of antifungal resistance in environmental yeasts, where FCZ resistance rates ranging from 20 to 35% have been described in urban aquatic systems.

For statistical comparisons, susceptibility outcomes were dichotomized as R versus NR (S + DD). Due to the presence of small sample sizes in some strata, Fisher’s exact test (two-sided) was applied, and effect sizes were expressed as OR with 95% CI (Table 8).

**Table 8:** Statistical comparison of antifungal resistance between groups

Comparison	Antifungal	OR	95% CI	*p* value
Urban vs. rural	ITZ	1.63	0.54–4.94	0.362
Urban vs. rural	FCZ	1.20	0.55–2.62	0.694
Winter vs. summer	ITZ	1.04	0.35–3.09	1.000
Winter vs. summer	FCZ	0.78	0.34–1.77	0.679
ITZ vs. FCZ	Overall	8.01	4.54–14.14	< 0.001

Spatial analysis indicated higher resistance frequencies in urban sites reaches compared to rural sections (Fig. 8). However, these differences were not statistically significant. For ITZ, urban site isolates showed increased odds of resistance relative to rural isolates (OR = 1.63; 95% CI: 0.54–4.94; *p* = 0.362). Similarly, FCZ R was slightly higher in urban sites (OR = 1.20; 95% CI: 0.55–2.62; *p* = 0.694). Although absolute resistance frequencies were consistently higher in urban samples, the lack of statistical significance suggests that variability and limited sample size may have reduced the power to detect differences between environments.

Seasonal analysis showed no significant differences in resistance patterns. For ITZ, resistance odds were comparable between winter and summer (OR = 1.04; 95% CI: 0.35–3.09; *p* = 1.000). For FCZ, a non-significant reduction in resistance was observed in winter (OR = 0.78; 95% CI: 0.34–1.77; *p* = 0.679). Although higher absolute numbers of resistant isolates were observed during winter, proportional analysis confirmed that seasonal variation did not significantly influence resistance distribution.

In contrast, resistance differed significantly between the antifungal agents. Resistance to ITZ was markedly higher than that to FCZ (OR = 8.01; 95% CI: 4.54–14.14; *p* < 0.001). This indicated substantially reduced susceptibility of environmental isolates to ITZ. The MIC_50_ and MIC_90_ values were 4 µg/ml and 8 µg/ml for ITZ, and 32 µg/ml and 64 µg/ml for FCZ, respectively. These values indicate the presence of yeast populations capable of growing at relatively high antifungal concentrations. However, MIC values cannot be directly compared between antifungal agents, as susceptibility classification depends on drug-specific interpretative breakpoints (Arendrup et al., 2020).

The higher frequencies of azole non-susceptible yeasts observed in urban river sections should be interpreted as an environmental pattern associated with anthropogenic pressure, rather than as direct evidence of causal selection mechanisms. Similar associations between wastewater discharge and reduced antifungal susceptibility have been reported in aquatic systems impacted by sewage and mixed effluents (Monapathi et al., 2017; Caicedo-Bejarano et al., 2023). Thus, the present findings are consistent with pollution-associated resistance patterns described in the literature, although causal relationships cannot be established based on the current dataset.

The higher resistance observed for itraconazole compared to fluconazole may be related to differences in environmental persistence and selective pressure. Azole compounds are widely used not only in clinical settings but also in agriculture, where triazole fungicides share structural similarities with medical antifungals (Toda et al., 2021). Environmental exposure to these compounds has been proposed as a driver of cross-resistance in *Candida* spp., potentially contributing to the enrichment of azole-tolerant populations in aquatic ecosystems (Bastos et al., 2021).

Although this study focused on phenotypic susceptibility, antifungal resistance in *Candida* spp. is often driven by genetic mechanisms. These include mutations in the *ERG11* gene and the overexpression of efflux pump genes (*CDR1*, *CDR2*, and *MDR1*), which are known to contribute to multidrug resistance (Chen et al., 2010; El-Kholy et al., 2023; Maheronnaghsh et al., 2022; Prasad et al., 2006). Future studies integrating molecular analyses and environmental parameters will be essential to better understand the drivers of antifungal resistance in aquatic ecosystems.

From a One Health perspective, the detection of antifungal-resistant yeasts in surface waters is of concern. These environments may act as reservoirs and dissemination pathways for resistance traits, potentially impacting human, animal, and environmental health.

#### Limitations and transferability

This study represents two seasonal sampling campaigns across four sites, and chemical contaminants such as antifungal residues were not quantified; therefore, causal relationships cannot be established. Additionally, yeast identification based on CHROMagar™ was intended for screening rather than definitive taxonomic classification. Despite these limitations, the combined use of conventional microbial indicators, yeast isolation, and standardized MIC profiling provides a low-cost and transferable framework for monitoring antifungal resistance in aquatic environments.

## Conclusion

This study demonstrated that urbanized sections of the Toledo River exhibited substantially higher microbial contamination and yeast densities compared to rural areas, with values reaching up to 351 CFU/100 ml. Antifungal susceptibility profiling revealed a predominance of azole non-susceptibility, particularly for itraconazole, with 44.3% of isolates classified as non-susceptible compared to 24.46% for fluconazole. While no spatial or seasonal differences were detected, resistance to itraconazole was significantly higher than to fluconazole (*p* < 0.001), indicating differential susceptibility among antifungal agents. The co-occurrence of elevated microbial indicators and azole non-susceptible yeasts in urban sites suggests a potential association with anthropogenic pressure, although causal relationships cannot be established. These findings support the role of aquatic environments as potential reservoirs of antifungal resistance and highlight the need to incorporate environmental compartments such as surface waters (rivers and lakes), wastewater treatment systems, sediments, and agricultural runoff into antimicrobial resistance surveillance frameworks. The integration of conventional microbial indicators, yeast abundance, and antifungal susceptibility profiling provides a comprehensive and transferable framework for monitoring water quality across diverse aquatic systems, including urban, peri-urban, and impacted rural environments. From a One Health perspective, this framework may contribute to improved environmental risk assessment and support strategies aimed at mitigating the dissemination of antimicrobial resistance. Future studies integrating chemical analyses and molecular approaches are essential to elucidate the mechanisms driving antifungal resistance in aquatic environments.

## Data Availability

All data generated or analyzed during this study are included in this published article and its supplementary information.
